# The relationship among college students’ mobile phone addiction, bedtime procrastination, and physical activity: a cross-lagged study

**DOI:** 10.3389/fpubh.2026.1831460

**Published:** 2026-06-11

**Authors:** Jiao Xu, Bo Li

**Affiliations:** 1 School of Sport and Health Sciences, Sichuan Technology and Business University, Meishan, Sichuan, China; 2 Department of Sports Training, Chengdu Sport University, Chengdu, Sichuan, China

**Keywords:** bedtime procrastination, college students, cross-lagged study, mobile phone addiction, physical activity

## Abstract

**Aim:**

In Chinese universities, mobile phone addiction (MPA) and bedtime procrastination (BP) are common and associated with adverse outcomes, whereas physical activity (PA) reliably benefits mental and physical health. Nevertheless, their longitudinal interplay remains underexplored. Accordingly, grounded in self-regulation theory, this longitudinal study examined the dynamic associations and potential causal pathways among MPA, BP, and PA in university students, in order to inform theory and guide future research.

**Methods:**

A total of 376 university students completed a valid questionnaire on problematic MPA, BP and PA at two points in time 3 months apart. A cross-lagged model was used to examine the longitudinal relationships between these factors.

**Results:**

Mobile phone addiction, BP, and PA exhibited complex temporal interrelations. Longitudinal path analyses indicated that: (1) mobile phone addiction (MPA), bedtime procrastination (BP) and physical activity (PA) all exhibited significant self-predictive effects between the two time points (MPA: *β* = 0.33, *p* < 0.05; BP: *β* = 0.28, *p* < 0.05; PA: *β* = 0.26, *p* < 0.01), indicating that these behavioral patterns exhibit a degree of stability; (2) There was a bidirectional reinforcement between MPA and BP, such that T1 MPA positively predicted T2 BP (*β* = 0.24, *p* < 0.001) and, reciprocally, T1 BP positively predicted T2 MPA (*β* = 0.20, *p* < 0.001); (3) PA functioned as a protective factor, whereby T1 PA predicted lower T2 MPA (*β* = −0.17, *p* < 0.001) and lower T2 BP (*β* = −0.19, *p* < 0.001); (4) Both MPA and BP exerted suppressive effects on PA, as T1 MPA (*β* = −0.25, *p* < 0.001) and T1 BP (*β* = −0.11, *p* < 0.001) negatively predicted T2 participation in PA.

**Conclusion:**

The findings suggest MPA intensifies BP and reduces PA engagement, potentially creating a self-perpetuating cycle. Therefore, promoting PA may counter both MPA and BP, providing empirical support for targeted university interventions on students’ phone use and sleep behaviors.

## 1 Introduction

As functionalities have diversified, mobile phones have evolved into indispensable tools for social connection, entertainment, work, shopping, and personal finance (1, 2). According to recent statistics, by December 2024 China counted 1.108 billion internet users, of whom 1.105 billion used mobile phones, yielding a 99.7% rate of mobile internet access (3). In this context, mobile phone addiction (MPA) has become a characteristic psychological-behavioral issue of the internet age, showing relatively high prevalence in youth populations. Empirical estimates place the prevalence of MPA among Chinese university students at 21.4% ~ 27.4% (4). Beyond impairing learning, daily functioning, and social relationships, MPA may have lasting negative consequences for both mental and physical health. In university samples, MPA frequently co-occurs with declines in academic achievement, social impairment, and mood fluctuations (5). At the same time, most undergraduates live outside the family context, affording greater freedom in smartphone use and fewer external constraints. Excessive phone use can trigger bedtime procrastination (BP), which in turn disrupts sleep routines and undermines sleep quality and next-day functioning among students. In a survey of 521 Chinese undergraduates, 96.08% reported pre-sleep phone use, with 71.90% persisting beyond 23:00 (6). Another separate survey of 815 adults indicated that risk of MPA exacerbated the problem, as individuals with addictive tendencies commonly reduced sleep to extend phone use (7). Related studies further suggest an association between MPA and BP (8). However, the majority of existing evidence is cross-sectional, leaving the bidirectional mechanisms and temporal dynamics underlying their interplay unclear. Accordingly, systematic, longitudinal investigation of the MPA-BP relationship is needed to clarify how MPA exerts its effects and to inform the development of effective interventions.

Physical activity (PA) is a proven health-promoting modality that enhances fitness and, importantly, holds promise as a component of intervention and treatment for MPA (9). Empirical work further suggests that PA can redirect students toward new interests and foster a more adaptive, health-promoting lifestyle, which in turn mitigates harms linked to MPA (10). Moreover, consistent with the sport socialization hypothesis—which views PA as a social context that fosters interpersonal adaptation (11, 12), behavioral and physiological findings show that PA strengthens inhibitory control and self-regulation (13), which may, in turn, lower procrastination (14). To integrate these observations, the present study draws on self-regulation theory. We propose that MPA depletes self-regulatory resources, which may increase bedtime procrastination and reduce physical activity, while sufficient physical activity can restore self-regulatory capacity and buffer against MPA and procrastination. To test these bidirectional processes, this longitudinal study examined the dynamic associations and potential causal directions among MPA, BP, and PA in university students, aiming to inform both theoretical refinement and evidence-based mental health practice on campuses.

## 2 Theoretical review and research hypotheses

### 2.1 Mobile phone addiction and bedtime procrastination

Mobile phone addiction (MPA) is a behavioral addiction, akin to internet or gaming addiction, arising from excessive use of modern technologies (15). Specifically, it refers to a compulsive pattern of phone overuse, driven by particular motives, that impairs users’ psychological and social functioning and induces anxiety and irritability upon temporary separation from the phone (16); in severe cases, physiological reactions such as numbness of the extremities, palpitations, dizziness, sweating, and gastrointestinal dysfunction may occur (17). Based on functional usage patterns, Tu et al. (18) classified MPA into three types: relational MPA (excessive reliance on phones for social interaction), entertainment MPA (overindulgence in phone-based entertainment), and information-seeking MPA (compulsive use of phones to search for information). MPA has emerged as a pressing societal concern, with particularly high salience in adolescent populations. Prior studies indicate a significant positive association between MPA and Bedtime procrastination—defined as delaying sleep despite intending to go to bed, negatively affects sleep quality and daily functioning (19). Social cognitive theory posits reciprocal interactions among behavior, cognition, and environment (20), implying that MPA may erode individuals’ self-control. Specifically, MPA can precipitate cognitive failures (21), impair attentional control (22), ultimately fostering procrastinatory behavior and degrading sleep quality (23). Related research further indicates that MPA-driven pre-sleep phone use gradually contributes to BP.

Meanwhile, self-regulation theory conceptualizes BP as a failure of self-control (24), thereby suggesting a tight connection between MPA and BP. Moreover, evidence indicates that both trait procrastination (including BP) and more generalized procrastination can precipitate MPA (25). Consistent with personality trait theory—which holds that traits shape behavioral tendencies (26), BP, as a procrastination-linked disposition, may likewise affect MPA. Empirical evidence also confirms that excessive phone use can entrap individuals in a vicious cycle of phone dependence and delayed bedtime (27). Taken together, we propose Hypothesis 1: among university students, MPA and BP mutually predict one another.

### 2.2 Mobile phone addiction and physical activity

Globally, smartphone addiction (MPA) is increasingly recognized as a public health issue, as both ownership and usage time keep climbing and multiple countries document rising addiction trends. Notably, the university years mark a transition in social adaptation, physical development, and psychological change and thus a key risk period for addiction (28), during which dependence on smartphones tends to be more salient. Reports indicate that approximately 59% of university students in Egypt exhibit some degree of smartphone dependence (29), whereas in China the proportion may reach about 80% (30). For students at this developmental juncture, overreliance on online engagement and immersion in virtual worlds can undermine physical and mental health, academic outcomes, and social relationships. For example, in a related study, Tufan et al. (31) found that MPA partially mediated the association between fear of missing out and phubbing behavior, while being phubbed moderated the effect of phubbing on friendship satisfaction, highlighting that MPA and related behaviors can have detrimental effects on university students’ interpersonal relationships (31).

Among available countermeasures, the intervention value of physical activity (PA) has garnered growing attention (32, 33). As an active lifestyle, PA promotes physical fitness, alleviates stress, enhances psychological resilience and executive function, and may also influence self-regulation. Empirical findings further suggest that well-dosed exercise, especially at moderate intensity, can ease off-phone discomfort and negative affect, reduce dependence on smartphones, and, in turn, ameliorate addiction-related problems (34, 35). However, through time displacement and attentional capture, MPA tends to compress the time and bandwidth available for engaging in PA (36). The bidirectional-effects hypothesis within self-regulation theory posits two pathways of mutual influence: (1) MPA consumes time resources and weakens momentary self-control, suppressing participation in PA; and (2) insufficient PA reduces self-control and accumulates negative affect, which in turn reinforces addictive behavior. Accordingly, MPA and PA may form a mutually reinforcing cycle (36). Thus, we advance Hypothesis 2: in university populations, MPA and engagement in physical activity are mutually influential, operating in both directions.

### 2.3 Bedtime procrastination and physical activity

Sufficient sleep is essential for individuals’ physical and mental health, academic performance, and overall development. However, the proliferation of mobile electronic devices and mounting academic pressure have led to widespread BP among university students (37). From the perspective of self-regulation theory, BP is considered a classic form of self-regulatory failure, i.e., a discrepancy between an individual’s bedtime intention and actual behavior that occurs in a state of depleted self-control resources (38). This behavior not only leads directly to sleep deprivation, difficulty initiating sleep, and daytime fatigue but may also, in the long term, trigger a range of psychophysiological health issues, including chronic sleep disorders, mood disturbances (e.g., anxiety, depression), and compromised immune function (39). Similarly, PA stands as a planned behavior that demands considerable self-control and energy (40). Consequently, the energy drain and routine disruption resulting from BP can directly undermine both the motivation and the ability to participate in PA. Prior research has confirmed that the trait of procrastination negatively predicts participation in PA (41). On the other hand, PA serves as an effective behavioral intervention that can exert a positive influence on procrastination. A substantial volume of empirical evidence indicates that regular PA boosts executive functions and self-control (42), which in turn negatively predicts general procrastinatory behaviors (43). Given the high degree of similarity in the psychological mechanisms of BP and general procrastination (19), it can be inferred that PA may reduce BP by enhancing self-regulatory resources, thus indirectly improving sleep quality. In sum, a review of the literature reveals that both PA and BP, as outcome variables in the Theory of Planned Behavior, are influenced by the theory’s core factors (44). This further suggests that they are not isolated behaviors but may have an interactive relationship. Therefore, we propose Hypothesis 3: A bidirectional relationship exists between university students’ bedtime procrastination and their engagement in physical activity.

**Figure 1:**
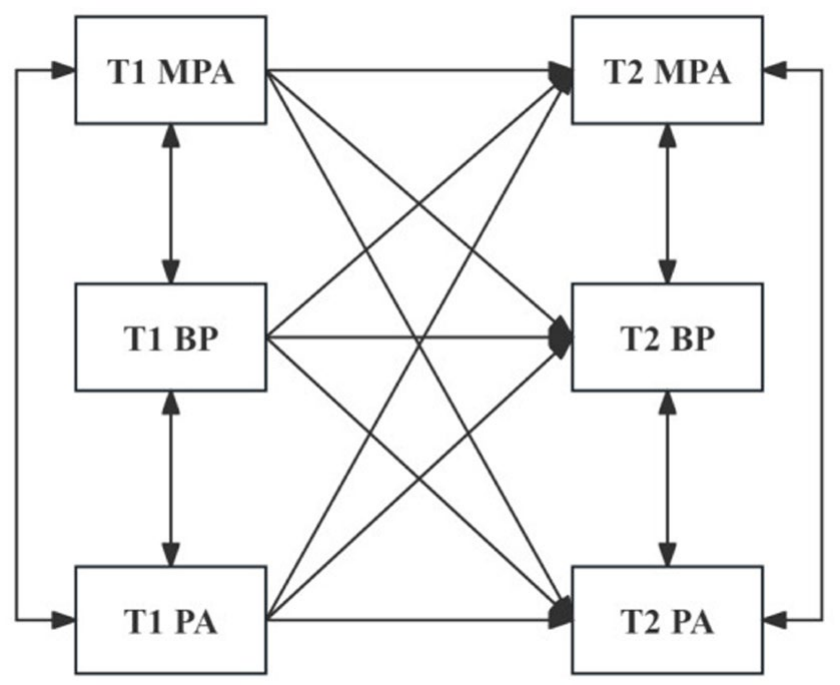
Model of conceptual framework. MPA, mobile phone addiction; BP, bedtime procrastination; PA, physical activity.

Building on this foundation, our study employs a longitudinal tracking cross-lag approach to construct a structural equation model (Figure 1) among college students, examining the causal relationships between MPA, BP, and PA. The research aims to reveal the underlying mechanisms of these behavioral patterns, providing practical guidance for mitigating smartphone addiction, fostering healthy sports attitudes, and promoting physical and mental well-being among university students.

## 3 Methods

### 3.1 Participants

We conducted a three-month longitudinal study, recruiting participants via a multi-stage cluster sampling approach from three universities situated in Sichuan Province and Chongqing, China. Prior to the survey administration, class counselors were briefed on the study’s objectives, and subsequently, all participating students gave their signed informed consent. Trained researchers conducted two waves of on-site data collection, administering the questionnaires to students as a group within their classrooms. A total of 416 questionnaires were distributed for this study. The first wave of data collection took place in February 2025 (T1), with 398 questionnaires returned, yielding a response rate of 95.7%; the second wave took place in May 2025 (T2), with 385 questionnaires returned, yielding a response rate of 92.5%. To ensure the accuracy of longitudinal data matching, this study used the last six digits of the student ID. The data cleaning procedure was as follows: (1) exclusion of invalid questionnaires containing pattern-based responses; (2) exclusion of questionnaires with missing or incorrectly entered last six digits of student ID; (3) exclusion of cases that participated in the survey at only one time point; (4) strict matching of T1 and T2 data based on student ID information, retaining only those questionnaires where the student IDs matched exactly across both measurements. Following the above quality control procedures, a final valid matched sample of 376 participants was obtained, representing a valid matching rate of 90.4%. In this study, errors in student ID entry (such as incorrect digit counts or transposed numbers) were regarded as random data entry errors with no theoretical association with core variables such as mobile phone addiction, bedtime procrastination or physical exercise behavior; therefore, the exclusion of these cases from the final sample is unlikely to introduce systematic bias number as the unique identifier linking the data from the two waves. Of these participants, 180 were male (47.9%) and 196 were female (52.1%). The average age for the final sample was 20.09 years (SD = 1.18).

### 3.2 Research tools

#### 3.2.1 Mobile phone addiction scale

The MPA scale used in this study was developed by Su et al. (45). This 22-item scale employs a 5-point Likert-type response format, where participants rate each statement on a scale from 1 (Very inconsistent) to 5 (Very consistent). It comprises six factors: withdrawal behavior, salience behavior, social comfort, negative consequences, app usage, and app updating. Higher total scores indicate a greater severity of smartphone addiction. The structural validity of this six-factor model has been previously established, with reports of good fit indices from confirmatory factor analyses (CFA). For our sample, the scale demonstrated excellent internal consistency at both waves of data collection, with Cronbach’s alpha coefficients of 0.943 (T1) and 0.948 (T2), respectively.

#### 3.2.2 Bedtime procrastination scale

To assess bedtime procrastination, this study used the Chinese version of the Bedtime Procrastination Scale (BPS), adapted by Zhang et al. (46) from the original scale by Kroese et al. (19). The scale includes 9 items rated on a 5-point Likert scale (1 = Never to 5 = Always), with four items being reverse-scored. Higher scores indicate a greater propensity for bedtime procrastination. The revision study reported a Cronbach’s *α* of 0.835, a test–retest reliability of 0.720, and high criterion validity.

#### 3.2.3 Physical activity rating scale

Physical activity was measured using the Physical Activity Rating Scale (PARS) developed by Liang (47), a widely used instrument for measuring exercise behavior among Chinese university students. The scale assesses three dimensions: intensity, duration, and frequency, with each rated on a 5-point Likert scale. The total physical activity score is the product of the scores from the three dimensions, which is then used to classify activity levels into low, medium, or high volume. In this study, Cronbach’s α coefficients were 0.686 (T1) and 0.728 (T2). The Kolmogorov–Smirnov test was significant (*p* < 0.001) for both time points.

### 3.3 Data analysis

Data processing and analysis were conducted using SPSS 27.0 and AMOS 26.0, with SPSS employed for common method bias testing, descriptive statistics, correlation analysis, independent samples t-tests, analysis of variance, and confirmatory factor analysis. AMOS was used for model construction and examination of variable relationships, including autoregressive coefficients and cross-lagged path coefficients (*β*). In these models, the variables were operationalized as: X1 representing smartphone addiction at T1 and X2 at T2; Y1 for bedtime procrastination at T1 and Y2 at T2; and Z1 for physical exercise behavior at T1 and Z2 at T2. To maintain the integrity of our findings, we selected control variables specifically to mitigate any potential confounding influences, thereby allowing for a clearer and more precise assessment of the relationships among the key variables. In this study, we incorporated gender, academic year (grade level), and field of study (major) as control variables, which helped to rule out their possible impacts on the observed outcomes.

### 3.4 Ethics statement

The study procedures were carried out under the ethical standards of the relevant national and institutional committees on human experimentation and with the Helsinki Declaration. We followed the Strengthening the Reporting of Observational Studies in Epidemiology (STROBE) guidelines. The study was approved by the Ethics Committee of Chengdu Sport University (Ethical Approval Number: 2025-13).

## 4 Results

### 4.1 Common method bias test

This study employed Harman’s single factor test to examine common method bias. At the first time point (T1), three factors with eigenvalues greater than 1 were extracted, with the first factor accounting for 39.845% of the total variance. At the second time point (T2), three factors with eigenvalues greater than 1 were also extracted, with the first factor explaining 36.035% of the total variance. Both time points fell below the 40% critical threshold. Therefore, severe common method bias was not present in either measurement of this study.

### 4.2 Descriptive statistics and correlations

Descriptive statistics and correlation analyses were conducted for MPA, BP, and PA among university students (Table 1). Correlation results indicated significant positive associations between smartphone addiction and bedtime procrastination at both time points (*p* < 0.01), significant negative associations between smartphone addiction and physical exercise behavior at both time points (*p* < 0.01), and significant negative associations between bedtime procrastination and physical exercise behavior at both time points (*p* < 0.01). At the same time, at time point T1, there were no significant differences between men and women in terms of mobile phone addiction (men: 67.43 ± 18.01; women: 68.35 ± 19.42), bedtime procrastination (men: 27.73 ± 7.99; females: 28.12 ± 7.83) and physical exercise behavior (males: 39.09 ± 31.76; females: 37.98 ± 30.86) (all *p* > 0.05). At time point T2, there were no significant differences between the two groups in mobile phone addiction (males: 68.12 ± 17.00; women: 68.23 ± 18.02), bedtime procrastination (men: 30.99 ± 7.86; women: 31.42 ± 7.62) and physical exercise behavior (men: 39.72 ± 29.45; females: 37.40 ± 28.35) were also not statistically significant (all *p* > 0.05) (Tables 2, 3).

**Table 1:** Mean, standard deviation and correlation analysis of mobile phone addiction, bedtime procrastination and physical activity behavior among university students.

Variable	T1 MPA	T1 BP	T1 PA	T2 MPA	T2 BP	T2 PA
T1 MPA	1					
T1 BP	0.276**	1				
T1 PA	−0.321**	−0.443**	1			
T2 MPA	0.441**	0.371**	−0.324**	1		
T2 BP	0.356**	0.296**	−0.335**	0.434**	1	
T2 PA	−0.229**	−0.209**	0.267**	−0.306**	−0.255**	1
M	67.91	27.93	38.52	68.18	31.21	38.52
SD	18.73	7.90	31.26	17.51	7.73	28.87

“**” indicates *p* < 0.01.

**Table 2:** Independent samples t-test for gender in T1, T2.

Grouping variable	Dependent variable	HV-test	Levene’s test for equality of variances		Mean equivalence *t*-test	
			*F*	*P*	*t*	*P*
Gender	T1MPA	Assume equal variance	1.621	0.204	−0.470	0.639
	T1BP	Assume equal variance	0.659	0.418	−0.477	0.633
	T1PA	Assume equal variance	0.162	0.687	0.343	0.732
	T2MPA	Assume equal variance	0.960	0.328	−0.058	0.954
	T2BP	Assume equal variance	0.367	0.545	−0.542	0.588
	T2PA	Assume equal variance	0.036	0.850	0.778	0.437

**Table 3:** Gender scores across various dimensions.

Gender	T1MPA	T1BP	T1PA	T2MPA	T2BP	T2PA
Male (*M* ± SD)	67.43 ± 18.01	27.73 ± 7.99	39.09 ± 31.76	68.12 ± 17.00	30.99 ± 7.86	39.72 ± 29.45
Female (*M* ± SD)	68.35 ± 19.42	28.12 ± 7.83	37.98 ± 30.86	68.23 ± 18.02	31.42 ± 7.62	37.40 ± 28.35

### 4.3 Cross-lagged model analysis

**Figure 2:**
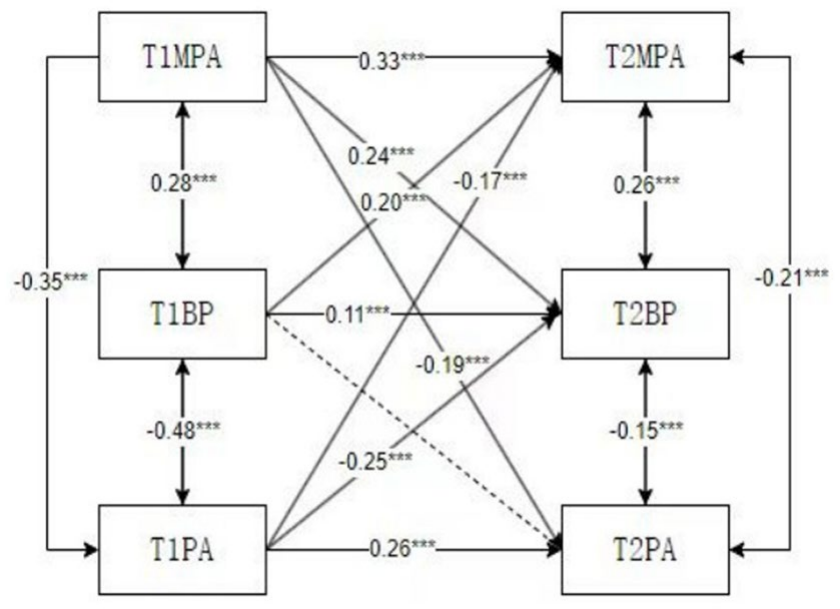
Cross-lagged analysis of mobile phone addiction, sleep procrastination and physical activity.

To investigate the dynamic, longitudinal relationships among mobile phone addiction (MPA), bedtime procrastination (BP), and physical activity (PA), a cross-lagged panel model was constructed using AMOS 26.0 (Model M1). Maximum likelihood estimation was employed. As detailed in Table 4, the structural equation model demonstrated an excellent fit to the data: *χ*^2^/df = 1.145, GFI = 0.985, CFI = 0.996, IFI = 0.996, TLI = 0.994, and RMSEA = 0.020. The standardized path coefficients for the cross-lagged model are illustrated in Figure 2. Analysis of the autoregressive paths revealed significant temporal stability across the three-month interval for MPA (*β* = 0.33, *p* < 0.001) and PA (*β* = −0.26, *p* < 0.001). Regarding the cross-lagged structural paths, the model revealed a bidirectional, reinforcing relationship between mobile phone addiction and bedtime procrastination. Specifically, T1 MPA positively predicted T2 BP (*β* = 0.24, *p* < 0.001), and reciprocally, T1 BP positively predicted T2 MPA (*β* = 0.20, *p* < 0.001). Furthermore, physical activity emerged as a significant protective factor against both negative behaviors. T1 PA negatively predicted subsequent levels of both T2 MPA (*β* = −0.17, *p* < 0.001) and T2 BP (*β* = −0.19, *p* < 0.001). Conversely, mobile phone addiction and bedtime procrastination exerted suppressive effects on future physical activity; both T1 MPA (*β* = −0.25, *p* < 0.001) and T1 BP (*β* = −0.11, *p* < 0.001) significantly and negatively predicted participation in PA at T2. Overall, this cross-lagged model unveils a complex cycle where screen time and sleep delay compound one another while simultaneously eroding physical activity engagement.

**Table 4:** Indicators of model fit.

Model name	$\chi^{2}/\mathrm{df}$	GFI	CFI	IFI	TLI	RMSEA
M1	1.145	0.985	0.996	0.996	0.994	0.020
standard	1–3	>0.900	>0.900	>0.900	>0.900	<0.10

## 5 Discussion

This study employed a cross-lagged design to examine the underlying relationships between mobile phone addiction, bedtime procrastination and physical activity among university students. Specifically, it was found that MPA and BP among university students could predict one another across time; MPA and PA could also predict one another across time; T1PA significantly predicted T2BP across time, whereas T1BP did not predict T2PA. This provides data to support further research into their longitudinal relationship. In addition, we observed no significant differences by gender or grade for any variable. One explanation may lie in sample homogeneity. All participants were Chinese university students who shared similar campus contexts, with convergent schedules and lifestyles. Regardless of gender or grade, students encounter comparable academic pressure, curricular structures, and social contexts, which could shape health-related metrics in similar ways (48). Additionally, in today’s Chinese university culture, attenuated gender role distinctions and broadly similar lifestyles may also help explain the lack of significant gender disparities. These findings provide new insight into the health of contemporary Chinese undergraduates and imply that university health interventions should prioritize individualized approaches over simple segmentation by gender or grade.

### 5.1 Mobile phone addiction and bedtime procrastination

Cross-lagged analyses indicated bidirectional predictive associations between MPA and BP, thereby supporting Hypothesis 1 and aligning with prior longitudinal evidence (49, 50). This pattern suggests a mutually reinforcing dynamic rather than a unidirectional causal chain. First, from the perspectives of sleep physiology and pre-sleep routines, nighttime smartphone use delays sleep onset and exacerbates BP through two pathways. One route is behavioral: phone use displaces the pre-sleep period, postpones going to bed, and provokes procrastination (80). The other route is physiological: screen-emitted light suppresses melatonin, prolongs sleep latency, and undermines sleep quality (51). Reliance on nighttime phone use for immediate gratification and stress relief substitutes short-term rewards for sleep benefits, fostering a habitual delay in bedtime. Over time, turning to the phone at night for instant relief and gratification can replace the long-term benefits of sleep, gradually (27). Second, drawing on cognitive—behavioral and coping theories, maladaptive beliefs are pivotal in both the emergence and persistence of addictive technology use (52). Under academic and social stress, students often regulate emotions or seek avoidance via phone entertainment, social media, or gaming (53–55), which consumes pre-sleep attentional and temporal resources and thereby erodes sleep and intensifies procrastination (56). Third, from immersion and self-regulation perspectives, phone use readily induces immersive states that distort time perception and weaken self-monitoring, delaying the intended bedtime (57). BP is conceptualized as a manifestation of self-regulatory failure, with higher BP linked to lower trait self-control and weaker executive functioning (24, 58, 59). Deficits in self-regulation also heighten vulnerability to uncontrolled phone use, thereby exacerbating real-world functional impairments and perpetuating a “insomnia—procrastination—overuse” cycle.

Evidence also supports the reverse direction of effects. BP prospectively predicts higher levels of problematic or addictive smartphone use. Surveys show that adolescents with high BP markedly increase phone use in the 3 h before bedtime and report more severe insomnia and later sleep onset (60). In middle-school samples, academic procrastination functions as an antecedent that promotes problematic phone use via mechanisms such as distraction cognitions (61, 62). Similarly, adult samples suggest mutual facilitation between procrastination and problematic new media use (63). Collectively, the evidence points to a plausible bidirectional causality between BP and MPA. The two may reinforce each other through maladaptive coping, immersive engagement, and deficits in self-regulation.

### 5.2 Mobile phone addiction and physical activity

This study found that MPA and PA can also predict each other across time. Specifically, higher prior MPA significantly and negatively predicted later PA, while higher prior PA significantly and negatively predicted later MPA. This finding supports our bidirectional hypothesis (H2) and aligns closely with the resource depletion and restoration perspectives of self-regulation theory. From the resource depletion pathway, MPA is often accompanied by negative affective states such as depression and anxiety, as well as social avoidance, which continuously deplete an individual’s self-regulatory resources (64). Since physical activity is a planned behavior requiring willpower, the cognitive capacity needed to initiate and sustain exercise markedly declines when self-control “reserves” are exhausted by MPA. According to the Uses and Gratifications Theory, smartphones reinforce high-frequency use by providing immediate entertainment and social gratification (65, 66). Such deep engagement directly crowds out time available for physical activity, fundamentally reducing opportunities for participation. Moreover, immersion in the virtual world diminishes the perceived value of real-world activities such as physical activity, thereby weakening intrinsic motivation to engage in these activities (67). This reliance on virtual instant gratification, in turn, exacerbates addictive behaviors (68). Conversely, from the resource restoration pathway, regular physical activity itself is a behavior that effectively restores and replenishes self-regulatory resources (69); through the neurophysiological and psychological restoration conferred by PA, individuals are better able to mobilize stronger inhibitory control when facing the temptation of mobile phone use (70), thereby reducing subsequent MPA. These two pathways act synergistically, constituting a negative spiral between MPA and PA: addiction undermines physical activity, while physical activity can in turn buffer against addiction. This bidirectional mechanism suggests that interventions targeting university student populations should simultaneously address reducing mobile phone dependency and promoting physical activity, so as to break the vicious cycle and establish a virtuous cycle to enhance overall well-being.

### 5.3 Physical activity and bedtime procrastination

The longitudinal results show that PA is a significant negative predictor of BP. Specifically, higher levels of PA engagement are associated with a lower frequency of BP behaviors. This finding provides strong support for the view of exercise as a tool for promoting self-regulation. The primary mechanism appears to be physiological: PA directly replenishes an individual’s self-regulatory resources by enhancing sleep quality. Abundant empirical evidence indicates that regular exercise significantly improves both subjective and objective sleep quality, optimizes sleep architecture, and reduces the incidence of sleep disturbances like BP (71, 72). From a neuroscientific standpoint, high-quality sleep is crucial for restoring metabolic activity in key brain regions such as the prefrontal cortex and limbic system, which serve as the neural substrates for executive functions (e.g., self-control, decision-making) (73). Sleep disruption caused by BP impairs this restorative process, thereby weakening next-day self-control and creating a vicious cycle: procrastination leads to poor sleep, which in turn weakens self-control and makes future procrastination even more likely.

In contrast, by promoting deep sleep, PA effectively facilitates the recovery of these neural systems, thereby enhancing the physiological and psychological capital needed to resist procrastination-related temptations at night and enabling individuals to better adhere to their intended bedtime (19). Second, PA may indirectly inhibit BP by strengthening an individual’s Future Time Perspective (FTP). As a cognitive disposition, FTP is an established protective factor against procrastination and a powerful driver of health-promoting behaviors, including PA (74, 75). Its mechanism lies in the fact that regular PA is itself a behavior oriented toward long-term health rewards. This behavioral pattern can subtly train individuals to prioritize long-term goals (e.g., next-day vitality, long-term health) over immediate gratification (e.g., late-night entertainment). Meta-analyses have confirmed that FTP is positively correlated with stronger self-control and lower impulsivity (76, 77). An individual with a future-oriented mindset is better able to leverage their rational decision-making framework to weigh costs and benefits and effectively inhibit the impulse to delay sleep, even when self-regulatory resources are depleted at night (78, 79). Therefore, it can be inferred that PA provides not only the physiological “energy” for self-control but also the cognitive “direction.” By fostering a mindset geared toward “investing in the future”, PA helps individuals consistently make choices at night that favor their long-term well-being, leading to a tangible reduction in bedtime procrastination.

## 6 Limitations

This study employed longitudinal tracking surveys and cross-lagged modeling to examine the dynamic relationships among MPA, BP, and PA in university students, offering insights for targeted interventions. However, this study has certain limitations: (1) At the methodological level, this study employs a traditional cross-lagged panel model for analysis. Although the CLPM is capable of revealing overall longitudinal patterns of association between variables, it fails to distinguish between the contributions of between-person stable traits and within-person dynamic fluctuations to cross-lagged effects. In recent years, the random intercept cross-lagged panel model (RI-CLPM) has been regarded as the standard method for such longitudinal studies, as it effectively decouples stable differences at the between-person level, thereby allowing for a more precise examination of causal effects at the within-person level. This study was limited to a two-time-point longitudinal design, whereas RI-CLPM typically requires three or more measurement waves to ensure the stability of model identification and parameter estimation; consequently, this method could not be adopted. Future research could employ a longitudinal design with at least three time points and utilize RI-CLPM to cross-validate the findings of this study, thereby further clarifying the causal relationships between MPA, BP, and PA at the within-person level. (2) This study employed only two time points, 3 months apart; whilst this is suitable for capturing short-term dynamics, it is difficult to reveal the longer-term evolution of relationships between the variables. Future research could extend the observation period and increase the frequency of measurements to examine the patterns of change in the relationship between the three variables at different stages of development. (3) The sample for this study was drawn from three universities across two provinces. Although the sample size met statistical requirements, the geographical coverage was limited; therefore, caution is advised when generalizing the findings to the national student population. Future research could involve multi-center collaboration, covering different geographical regions and institutions of varying levels across China, in order to verify the geographical generalisability of the findings from this study. (4) All variables in this study were measured using self-report methods. Although Harman’s one-factor test did not reveal any significant common-method bias, future research could incorporate objective measurement tools (such as app-recorded mobile phone usage duration and sleep and exercise data recorded by wearable devices) to enhance the objectivity of the data. (5) Furthermore, the Cronbach’s alpha coefficient for the Physical Activity Scale at time point T1 was 0.686, which is slightly below the conventional threshold of 0.70. This scale measures total physical activity as the product of exercise intensity, duration and frequency; its structure is essentially formative in nature, and internal consistency is not the optimal criterion for assessing its reliability. However, lower measurement reliability may lead to an underestimation of the true relationship between variables, resulting in a conservative estimation of the strength of the association between physical exercise and mobile phone addiction and bedtime procrastination in this study. The improvement in the *α* coefficient (0.728) at the T2 time point suggests that the measurement quality of this scale has improved with repeated administration. Future research could combine objective measurement tools (such as accelerometers and fitness trackers) with standardized exercise diaries to address the reliability limitations of self-report scales.

## 7 Conclusion

This study demonstrates that MPA not only exacerbates BP but may also reduce individuals ‘likelihood of engaging in PA, creating a self-reinforcing cycle. PA serves as a protective factor against MPA and BP. The findings provide empirical support for universities to develop targeted intervention strategies, highlighting the positive impact of promoting physical exercise in improving college students’ mobile phone usage habits and sleep behaviors.

## Data Availability

The raw data supporting the conclusions of this article will be made available by the authors, without undue reservation.
